# An Effective Electrodeposition Mode for Porous MnO_2_/Ni Foam Composite for Asymmetric Supercapacitors

**DOI:** 10.3390/ma9040246

**Published:** 2016-03-30

**Authors:** Yi-Chiun Tsai, Wein-Duo Yang, Kuan-Ching Lee, Chao-Ming Huang

**Affiliations:** 1 Department of Chemical and Materials Engineering, National Kaohsiung University of Applied Sciences, Kaohsiung 80778, Taiwan; james810221@gmail.com (Y.-C.T.); ywd@cc.kuas.edu.tw (W.-D.Y.); 2 Department of Materials Engineering, Kun Shan University, Tainan 71070, Taiwan; z819002z@gmail.com

**Keywords:** electrodeposition, MnO_2_, Ni foam, asymmetric supercapacitor

## Abstract

Three kinds of MnO_2_/Ni foam composite electrode with hierarchical meso-macroporous structures were prepared using potentiodynamic (PD), potentiostatic (PS), and a combination of PS and PD(PS + PD) modes of electrodeposition. The electrodeposition mode markedly influenced the surface morphological, textural, and supercapacitive properties of the MnO_2_/Ni electrodes. The supercapacitive performance of the MnO_2_/Ni electrode obtained via PS + PD(PS + PD(MnO_2_/Ni)) was found to be superior to those of MnO_2_/Ni electrodes obtained via PD and PS, respectively. Moreover, an asymmetric supercapacitor device, activated carbon (AC)/PS + PD(MnO_2_/Ni), utilizing PS + PD(MnO_2_/Ni) as a positive electrode and AC as a negative electrode, was fabricated. The device exhibited an energy density of 7.7 Wh·kg^−1^ at a power density of 600 W·kg^−1^ and superior cycling stability, retaining 98% of its initial capacity after 10,000 cycles. The good supercapacitive performance and excellent stability of the AC/PS + PD(MnO_2_/Ni) device can be ascribed to its high surface area, hierarchical structure, and interconnected three-dimensional reticular configuration of the nickel metal support, which facilitates electrolyte ion intercalation and deintercalation at the electrode/electrolyte interface and mitigates volume change during repeated charge/discharge cycling. These results demonstrate the great potential of the combination of PS and PD modes for MnO_2_ electrodeposition for the development of high-performance electrodes for supercapacitors.

## 1. Introduction

There is increasing demand for electrical energy storage for hybrid electric vehicles, uninterruptible power supplies, and mobile electronic devices. Supercapacitors exhibit high power density, fast charging, and excellent cycling stability, making them a promising candidate for next-generation power devices [1,2,3]. Based on the charge storage mechanism, supercapacitors can be classified into two broad categories: electrical double-layer capacitors and pseudocapacitors [4,5]. Carbon-based materials are frequently used as electrode materials for double-layer capacitors due to their long life-cycles and great mechanical properties [6,7]. However, the capacitance of double-layer capacitors is limited by the electrical charge accumulation at the interface between the electrode and electrolyte. Transition metal oxides and conducting polymers are commonly used as electrode materials for pseudocapacitors [8,9,10]. Based on the pseudocapacitive energy storage mechanism, metal oxides/hydroxides, such as TiP_2_O_7_ [11], NiO/RuO_2_ [12], LiTi_2_(PO_4_)_3_ [13], and Nb_2_O_5_ [14], and metal nitrides MN (M = Cr, Co) [15], MgCo_2_O_4_ [16], and Zn(OH)^2−^–4Zn(OH)_4_^2−^ [17] can provide higher specific capacitance than that provided by conventional carbon materials and better cycling stability than that of polymer materials. By incorporating carbon materials and transition metal oxides in an asymmetric supercapacitor, the capacitance of a supercapacitor can be significantly enhanced [18]. Recently, metal oxides and oxysalts as anode materials for Li-ion batteries have been introduced to electrochemical supercapacitors [19].

Among various transition metal oxides used for supercapacitors, manganese oxide is a promising material due to its high theoretical specific capacitance, low cost, environmental friendliness, and natural abundance [20,21,22]. Traditionally, active MnO_2_ powder is mixed with a conductive agent and a polymer binder into a paste, which is then coated onto a substrate as an electrode [23,24,25,26,27]. However, the overall capacity and the volumetric capacity of the electrode are significantly sacrificed due to the usage of large amounts of binder and conductive agent during electrode fabrication [28]. Moreover, the weak bond between MnO_2_ and the current collector may deteriorate during charge-discharge cycles, resulting in poor cycle life. Electrodeposition is a low-cost, low-temperature process that allows large-scale deposition and direct control of film thickness without a binder for the fabrication of porous electrodes with various morphologies, achieved via control of the deposition bath and deposition conditions [29,30,31]. Electrodeposition can be generally divided into galvanostatic (GS), potentiodynamic (PD), potentiostatic (PS), and pulsed deposition (pulsed current and pulsed potential) techniques according to the type of applied voltage or current [32]. Dubal [29] deposited MnO_2_ films onto stainless steel foil using three electrodeposition modes, namely PD, PS, and GS, and found that MnO_2_ films obtained using PD had a higher specific capacitance (237 F·g^−1^) than those of films obtained using PS and GS (196 and 184 F·g^−1^, respectively). To the best of our knowledge, no previous work has compared the supercapacitive performance of MnO_2_ electrodes obtained using PD, PS, and a combination of PS and PD modes (PS + PD). The optimal electrodeposition mode for the preparation of MnO_2_ electrodes for supercapacitors has not yet been determined. It is believed that an electrode with a porous structure and a high specific surface area is particularly effective in enhancing the electrochemical properties of supercapacitors. As a substrate for supercapacitors, nickel (Ni) metal foam has several advantages, including low cost, a three-dimensional (3D) reticular configuration, high porosity, and high specific surface area [33].

In this study, nanostructured porous MnO_2_/Ni foam composites were prepared via three electrodeposition modes and used as the positive electrode of an asymmetric supercapacitor. Through the electrodeposition method, the MnO_2_ film was directly grown on the backbone of the 3D Ni foam without a polymer binder, resulting in a reduction of the contact resistance between the MnO_2_ and Ni foam and a higher transport rate of ions/electrons. The structure, morphology, and electrochemical properties of as-prepared MnO_2_/Ni composites were investigated. The MnO_2_/Ni obtained via a combination of PS and PD modes (PS + PD) had a hierarchical structure, which increased the electrochemically active surface area. The as-prepared PS + PD(MnO_2_/Ni) exhibited a promising specific capacitance. Furthermore, an asymmetric supercapacitor composed of a PS + PD(MnO_2_/Ni) positive electrode and an activated carbon (AC) negative electrode was fabricated. It exhibited a large specific capacitance, a high energy density, and excellent cycling performance.

## 2. Experimental Section

### 2.1. Preparation of MnO_2_/Ni Foam Composites

Manganese oxide films were deposited onto Ni foam using PD, PS, and PS + PD electrodeposition techniques, respectively. Before deposition, Ni foam (ChangSha Lyrun New Material Co. Ltd., Changsha, China, 110 pores per square inch) was ultrasonically degreased in acetone for 30 min, treated with 2 M HCl for 20 min, washed with distilled water, and dried in an oven at 80 °C overnight. An aqueous precursor solution with 0.1 M Mn(CH_3_COO)_2_ and 0.1 M Na_2_SO_4_ was used as the electrolyte. All depositions were carried out at room temperature with a three-electrode setup, where Ni foam, Pt foil, and a saturated calomel electrode (SCE) were used as the working, counter, and reference electrodes, respectively. The PD deposition of MnO_2_ was conducted in the potential range of +0.3 to +0.6 V (*vs.* SCE) at a scan rate of 25 mV·s^−1^ for 1500 cycles. PS deposition was carried by applying a voltage of +0.6 V (*vs.* SCE) for 1800 s. For PS + PD, PS deposition (+0.6 V (*vs.* SCE) for 900 s) was conducted, followed by PD deposition (+0.3 and +0.6 V (*vs.* SCE) at a scan rate of 25 mV·s^−1^ for 800 cycles). After deposition, the film was thoroughly washed with deionized water, dried, and annealed at 300 °C for 2 h in air. The amount of manganese oxide deposited onto the Ni foam substrate was evaluated by calculating the weight difference of the working electrodes. The mass loading of the manganese oxide deposited using PD, PS, and PS + PD was about 4 mg. In this work, the manganese oxide films electrodeposited onto Ni foam via PD, PS, and PS + PD modes are denoted as PD(MnO_2_/Ni), PS(MnO_2_/Ni), and PS + PD(MnO_2_/Ni), respectively. 

### 2.2. Sample Characterization

The X-ray diffraction (XRD) patterns of the obtained samples were measured using an X-ray diffractometer (PANalytical X’Pert PRO, Eindhoven, The Netherland) with Cu radiation (λ = 0.15418 nm) in the 2θ range of 20°–60°. The surface morphology was examined using scanning electron microscopy (SEM, JEOL JSM-6700F, Peabody, MA, USA). The surface area and pore volume of the MnO_2_ powders, scratched from the MnO_2_/Ni composite, were determined using a volumetric sorption analyzer (Micromeritics ASAP 2020, Norcross, GA, USA).

### 2.3. Electrochemical Measurements of Single Electrode and Asymmetric Supercapacitor

For the single electrode, cyclic voltammetry (CV), galvanostatic charge/discharge (GCD), and electrochemical impedance spectroscopy (EIS) measurements were conducted at room temperature to assess the electrochemical properties of the electrodes and fabricated asymmetric supercapacitor. The CV and EIS measurements were carried out on an electrochemical workstation (CH Instruments, CHI 660B). In half-cell tests, the electrochemical tests of MnO_2_/Ni electrodes were conducted in a three-electrode glass cell with 9 M LiNO_3_ as the aqueous electrolyte. Platinum foil and SCE electrodes were used as counter and reference electrodes, respectively. The MnO_2_/Ni electrodes served directly as the working electrodes. The mass of MnO_2_ active material was ~4 mg·cm^−2^. For the cyclic voltammograms, the sweep rate was set at 5 mV·s^−1^ with a potential window of 0–1.2 V *vs.* the SCE reference electrode. The average specific capacitance of the MnO_2_/Ni electrodes can be calculated from the CV curves by integrating the area under the current-potential curve:

(1) $$C=\frac{1}{m\nu (V_{c}-V_{a})}\int_{V_{a}}^{V_{c}}I(V)dV$$

where *C* is the specific capacitance (F·g^−1^), *m* is the mass of the active material in the electrode, *ν* is the potential scan rate (mV·s^−1^), *V_c_* − *V_a_* is the potential window, and *I*(*V*) is the response current (mA).

In full-cell tests, a rectangle-type asymmetric supercapacitor was assembled with the MnO_2_/Ni electrode as the positive electrode, AC as the negative electrode, polypropylene as the separator, 9 M LiNO_3_ as the electrolyte, Ni mesh as the collector, and laminated aluminum foil as the outer package. The AC electrode was prepared by coating an *N*-methyl pyrrolidone (NMP)-based slurry made of coconut carbon, pre-oxidized by HNO_3_, with a Brunauer-Emmett-Teller (BET) surface area of 820 m^2^·g^−1^, PVDF, super-P carbon, and KS-6 carbon (75:10:10:5) onto Ni foam, which was subsequently dried at 110 °C for 1 h under vacuum conditions and then pressed at 10.0 MPa for 10 s. The mass ratio of the positive electrode to the negative electrode was 1:2. The GCD performance of the asymmetric supercapacitor was evaluated using a battery testing station (Scribner Associates Inc., Southern Pines, NC, USA, 580) in the range of 0–1.2 V at a constant current of 1 A·g^−1^. The specific capacitance, *C*, was calculated using the equation *C* = (*I* × Δ*t*)/(Δ*V* × *m*), where *C* is the specific capacitance of the active materials, *I* is the discharging current, Δ*t* is the discharging time, Δ*V* is the discharging potential difference, and *m* is the mass of the loaded active materials. The EIS measurements were obtained in the frequency range of 0.01 Hz–100 kHz with a perturbation amplitude of 5 mV *vs.* the open-circuit potential. The energy density (*E*) of the asymmetric supercapacitor can be calculated from the specific capacitance (*C*) and the cell voltage (*V*) as:

*E* = 0.5 × *CV*^2^ (Wh·kg^−1^) (2)

The power density (*P*) of the asymmetric supercapacitor can be calculated from the energy density (*E*) and the discharging time (*t*) as:

*P* = *E*/*t* (W·kg^−1^) (3)

## 3. Results and Discussion

### 3.1. Sample Characterization

#### 3.1.1. Structure Analysis

**Figure 1:**
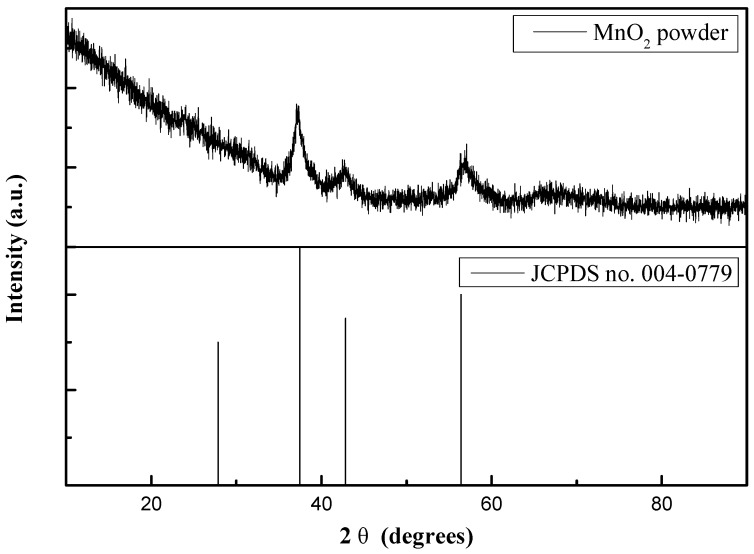
XRD pattern of PS + PD(MnO_2_/Ni).

The structural investigation of the as-prepared MnO_2_/Ni composite electrodes was conducted using XRD analysis. No distinct diffraction peaks of MnO_2_ were observed in the XRD patterns (not shown); only those corresponding to the Ni foam substrate appeared. To avoid the effect of Ni foam, XRD was measured from MnO_2_ powders scratched from MnO_2_/Ni composite electrodes. As shown in Figure 1, the XRD pattern of scraped MnO_2_ powder deposited using PS + PD mode exhibited three significant peaks and can be indexed as pyrolusite MnO_2_ (JCPDS card No. 004-0779) [34]. These peaks were broad and weak, indicating that the MnO_2_ obtained via PS + PD was nanocrystalline in nature.

#### 3.1.2. SEM Characterization of Electrodes

**Figure 2:**
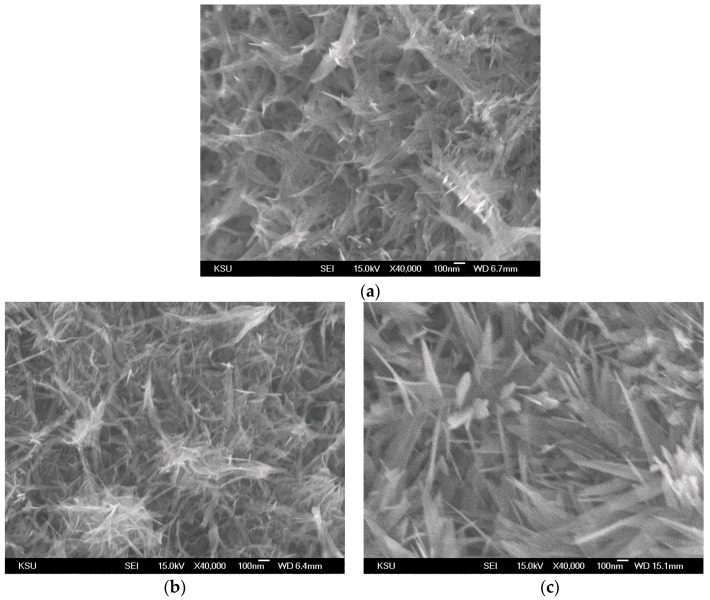
SEM images of (**a**) PD(MnO_2_/Ni); (**b**) PS(MnO_2_/Ni); and (**c**) PS + PD(MnO_2_/Ni).

Figure 2 shows SEM images of MnO_2_/Ni composite electrodes deposited using PD, PS, and PS + PD, respectively. The morphology of the prepared samples strongly depends on the electrodeposition method. As shown in Figure 2a, MnO_2_ was deposited uniformly over the skeleton of the Ni foam substrate and a large number of nanosheets aggregated to form a 3D network when PD mode was used. From Figure 2b, the surface morphology of the prepared sample changed from nanosheets to nanowires. The length of the nanowires was up to over 1 μm and the diameter was about 2–5 nm. For PS + PD mode, Figure 2c, MnO_2_ nanosheet arrays with sharp tips uniformly covered the surface of the Ni foam. All of the MnO_2_ nanosheets had a tapered morphology toward their tips, forming a triangular outer shape; the bottom width was less than 100 nm and the tip width was about 10 nm. Moreover, the thickness of the nanosheets was in the range of 2–5 nm. With PS and PS + PD modes, the obtained highly-porous structures provide plenty of space for the transport of the electrolyte into the electrode material, which is of great importance for effectively utilizing electro-active materials and achieving excellent electrochemical performance. The morphological characteristics of the grown MnO_2_/Ni composite electrodes can be controlled by adjusting the deposition route.

#### 3.1.3. Porosity and Surface Area Characterization

**Figure 3:**
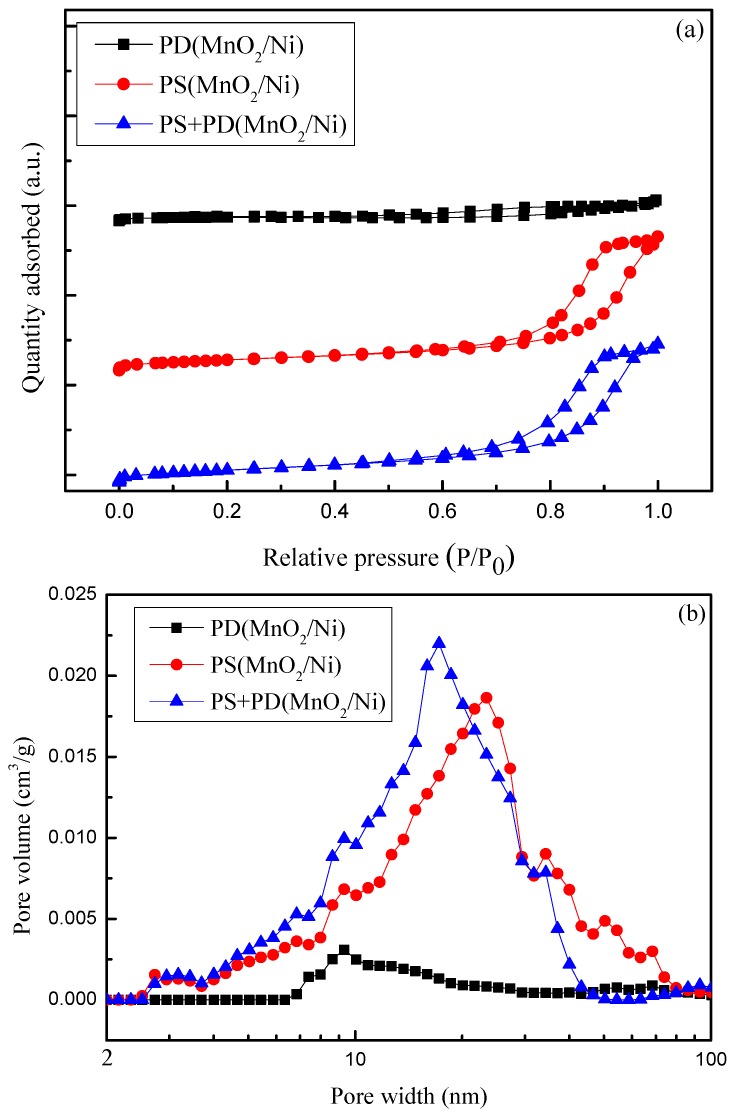
(**a**) N_2_ adsorption/desorption isotherms; and (**b**) corresponding pore size distributions of PD(MnO_2_/Ni), PS(MnO_2_/Ni), and PS + PD(MnO_2_/Ni).

Nitrogen adsorption-desorption isotherms and the corresponding pore-size distributions of the as-prepared samples are shown in Figure 3. Type IV isotherms with hysteresis loops can be seen in Figure 3a, demonstrating that all electrodeposited MnO_2_/MF samples had a typical mesoporous structure. The N_2_ adsorption isotherms of PS(MnO_2_/Ni) and PS + PD(MnO_2_/Ni) show a very slow increase in N_2_ adsorption up to 0.90 of the relative pressure (*P*/*P*_0_), where a steep increase of the adsorbed volume was observed and capillary condensation took place. The triangular shape and a steep desorption branch of the isotherms forms a H_2_-type hysteresis loop, suggesting the presence of highly interconnected pores with narrow mouths and wider bodies (ink-bottle-like pores) [35]. As shown in Figure 3b, the mesopores of PD(MnO_2_/Ni) have a distribution centered at around 9 nm, with a small portion expanding into macropores. A wide range of sizes that cross the mesopore-macropore boundary and expand into macropores was observed for PS(MnO_2_/Ni). PS(MnO_2_/Ni) and PS + PD(MnO_2_/Ni) exhibit highly porous structures with pore diameters ranging from 3 to 70 nm, with pore size distributions centered at 25–50 nm and 18–35 nm, respectively, suggesting hierarchical porous structures. The specific surface area, calculated using the BET equation, pore volume, and average pore diameter, obtained via the Barrett-Joyner-Halenda (BJH) equation using the adsorption isotherm branch, are listed in Table 1. The BET specific surface areas of PD(MnO_2_/Ni), PS(MnO_2_/Ni), and PS + PD(MnO_2_/Ni) samples are 21, 86, and 103 m^2^·g^−1^, respectively; the pore volumes of PS(MnO_2_/Ni) and PS + PD(MnO_2_/Ni) are larger than that of PD(MnO_2_/Ni). Analyses of the pore size distribution reveal that the BJH adsorption average pore diameter values of PD(MnO_2_/Ni), PS(MnO_2_/Ni), and PS + PD(MnO_2_/Ni) samples are 8, 14, and 13 nm, respectively. The difference in texture properties of the MnO_2_/Ni samples is in good agreement with the surface morphology results. The hierarchical porous structures of PS(MnO_2_/Ni) and PS + PD(MnO_2_/Ni) provide efficient transport for electrons and ions, making a significant contribution to a high electrochemical capacity. It has been reported that the ideal electrode material should have a hierarchical porous structure consisting of macropores (larger than 50 nm) for the ion-buffering reservoir, mesopores (2–50 nm) for ion transport, and micropores (less than 2 nm) for charge storage [36].

### 3.2. Electrochemical Performances of MnO_2_/Ni Single Electrode

**Figure 4:**
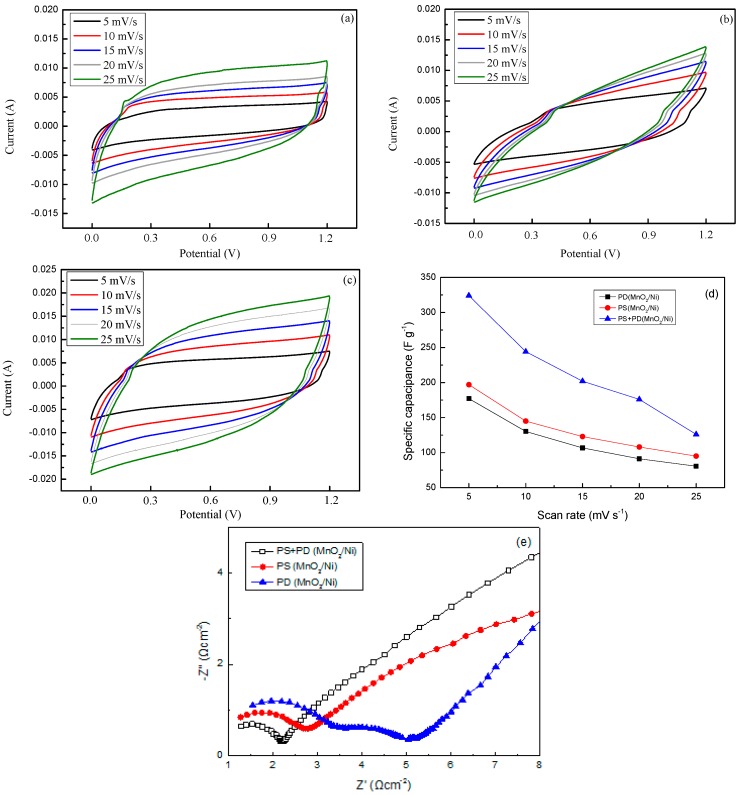
Cyclic voltammograms of (**a**) PD(MnO_2_/Ni); (**b**) PS(MnO_2_/Ni); (**c**) PS + PD(MnO_2_/Ni) electrodes obtained at various scan rates; and (**d**) plots of specific capacitance *vs.* potential scan rate; and (**e**) Nyquist plots of PD(MnO_2_/Ni), PS(MnO_2_/Ni), and PS + PD(MnO_2_/Ni) electrodes.

Before the MnO_2_/Ni composite electrode was employed for asymmetric supercapacitor fabrication, CV and EIS measurements of MnO_2_/Ni electrodes obtained using the three deposition modes were performed to investigate their capacitive behavior and ion transport properties. The CV studies of the obtained MnO_2_/Ni electrodes were carried out in 9 M LiNO_3_ electrolyte within 0 to +1.2 V *vs.* SCE operational windows, respectively, at various scan rates (5–25 mV·s^−1^) using a three-electrode cell configuration. The current density of the MnO_2_/Ni electrodes increased with increasing scan rate, indicating excellent supercapacitive behavior for all electrodes (Figure 4a–c). The CV curves of the MnO_2_/Ni electrodes have a quasi-rectangular shape at all scan rates, indicating that the capacitance characteristics of the electrodeposited MnO_2_ oxide electrodes are different from those of electric double-layer capacitance, whose CV curve has a nearly ideal rectangular shape. The quasi-rectangular shape of the CV profile is associated with the reversible successive surface redox reactions of MnO_2_, the oxidation from Mn(III) to Mn(IV), and the reduction from Mn(IV) to Mn(III) [37]. However, the CV curve deviated obviously from its quasi-rectangular shape when the applied scan rate was increased to 20–25 mV·s^−1^, which was due to the kinetics of electron transport in the electrode materials and the limited ion adsorption-desorption process at the interface of the electrode and electrolyte. Of note, the current density and CV curve integral areas of the PS + PD(MnO_2_/Ni) electrode are much larger than those of PD(MnO_2_/Ni) and PS(MnO_2_/Ni) electrodes, indicating a larger capacitance of the PS + PD(MnO_2_/Ni) electrode. The specific capacitances *vs.* potential scan rate of the MnO_2_/Ni electrodes are shown in Figure 4d. The PS + PD(MnO_2_/Ni) electrode shows the highest capacitance among all electrodes from high to low scan rates. The capacitance values of PD(MnO_2_/Ni), PS(MnO_2_/Ni), and PS + PD(MnO_2_/Ni) electrodes are 175, 200, and 325 F·g^−1^, respectively, for a scan rate of 5 mV·s^−1^. The capacitance of PD(MnO_2_/Ni) decreased to 75 F·g^−1^ when the scan rate was increased to 25 mV·s^−1^. However, PS + PD(MnO_2_/Ni) retained a capacitance of 125 F·g^−1^ at this scan rate, which is nearly 1.7 times that of PD(MnO_2_/Ni). The BET and CV results indicate that the superior pseudocapacitive performance of PS + PD(MnO_2_/Ni) is due to its unique hierarchical porous structure. The existence of an interconnected porous structure whose walls possess finer pores leads to enhanced mass transport through the former and high specific surface area due to the latter. A larger electrode/electrolyte contact area and a shorter diffusion length of Li^+^ ions can be obtained in a hierarchical porous structure, leading to lower inner resistance, which is beneficial for higher specific capacity.

EIS was carried out to further analyze the electrochemical behaviors of the deposited MnO_2_/Ni electrodes. EIS offers information regarding the internal resistance of the electrode material and the resistance between the electrode and electrolyte. Figure 4e shows the Nyquist plots of the PD(MnO_2_/Ni), PS(MnO_2_/Ni), and PS + PD(MnO_2_/Ni) electrodes measured in the frequency range of 0.01–100 kHz with a perturbation amplitude of 5 mV *vs.* the open-circuit potential, where *z*′ and *z*′′ are the real and imaginary parts of the impedance, respectively. As can be seen, the Nyquist plot for each sample comprises a high-frequency semicircle and a low-frequency straight line. The diameter of the semicircle in the high-frequency range is associated with the charge transfer resistance; the diameter of the semicircle of the PS + PD(MnO_2_/Ni) electrode is smaller than those of PD(MnO_2_/Ni) and PS(MnO_2_/Ni) electrodes (the estimated resistance values are 0.9, 1.9, and 1.9 Ω, respectively), suggesting a lower charge-transfer resistance of PS + PD(MnO_2_/Ni). These results are in accordance with the CV data, with the PS + PD(MnO_2_/Ni) electrode exhibiting the best supercapacitor performance. The slope of the curve in the low-frequency region is the Warburg impedance (*Z_w_*) and reflects the diffusive resistances, including electrolyte diffusion and proton diffusion, in the host materials. The higher the angle of the line, the more closely the capacitor behaves as an ideal supercapacitor. The impedance slope of PS + PD(MnO_2_/Ni) has a high angle (above 45°), indicating a high mobility of Li^+^ ions in the PS + PD(MnO_2_/Ni) electrode. Both the charge-transfer resistance and diffusive resistance of PS + PD(MnO_2_/Ni) are lower than those of PD(MnO_2_/Ni) and PS(MnO_2_/Ni) electrodes, which is attributable to the effective porous structure of PS + PD(MnO_2_/Ni) facilitating electron and ion transport.

### 3.3. Electrochemical Performance of AC/(MnO_2_/Ni) Asymmetric Supercapacitor Devices

**Figure 5:**
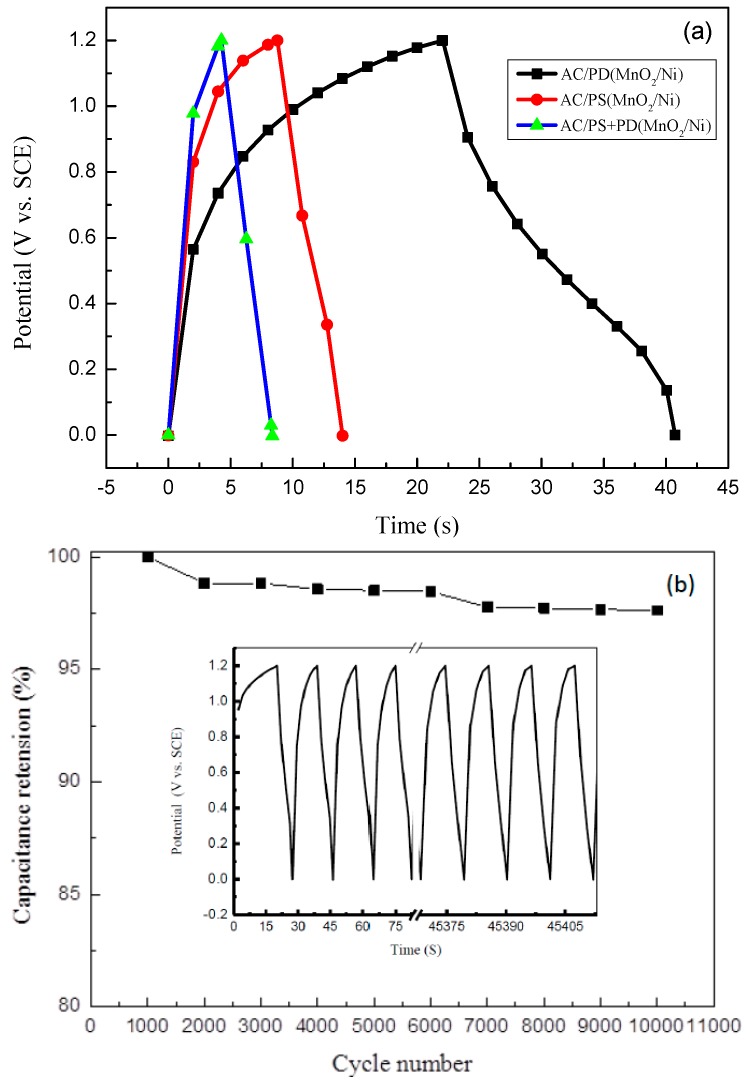
Performance of asymmetric AC/MnO_2_ device using MnO_2_/Ni as positive electrode and AC as negative electrode. (**a**) Charge-discharge curves of AC/PD(MnO_2_/Ni), AC/PS(MnO_2_/Ni), and AC/PS + PD(MnO_2_/Ni); and (**b**) cycling stability test of AC/PS + PD(MnO_2_/Ni) device.

To further investigate the capacitive performance of MnO_2_ arrays on Ni metal foam deposited using the three deposition modes, asymmetric supercapacitor devices were assembled using MnO_2_/Ni and the coconut-based AC as the positive and negative electrodes, respectively (denoted as AC/MnO_2_). The GCD tests were performed for the obtained AC/PD(MnO_2_/Ni), AC/PS(MnO_2_/Ni), and AC/PS + PD(MnO_2_/Ni) devices. The tests were carried out in a 9 M LiNO_3_ aqueous electrolyte under a constant charge and discharge current density of 1 A·g^−1^. As shown in Figure 5a, the first cycle of the nonlinear charge/discharge curves confirms the pseudo-capacitive behavior of all devices. In this study, the discharge profile of an asymmetric supercapacitor device consists of three regions: a rapid drop of voltage due to the internal resistance of MnO_2_, a linear deviation of the time dependence of the potential related to the double-layer capacitance behavior, and slope variation of the time dependence of the charge transfer reaction of MnO_2_ related to pseudo-capacitance behavior, resulting from the electrochemical adsorption/desorption or redox reaction at the interface between the electrode and electrolyte [38]. Moreover, the time required for charging and discharging was highest for AC/PS + PD(MnO_2_/Ni), which has higher specific energy and power density than those of AC/PD(MnO_2_/Ni) and AC/PS(MnO_2_/Ni). The specific energy and specific power of AC/PD(MnO_2_/Ni), AC/PS(MnO_2_/Ni), and AC/PS + PD(MnO_2_/Ni) devices are listed in Table 2.

The long-term cycling stability of a supercapacitor device is an essential requirement for energy storage. The AC/PS + PD(MnO_2_/Ni) device with the highest capacitance was chosen for the evaluation of the cycling stability through repeated charging and discharging measurements at a constant current density of 1 A·g^−1^ , in the potential range of 0 to 1.2 V. As shown in Figure 5b, the AC/PS + PD(MnO_2_/Ni) device exhibited stable cycling performance and 98% of its initial specific capacitance was retained after 10,000 cycles. As shown in the inset of Figure 5b, the charge/discharge times of 9993–10,000 cycles are almost the same as those for the first five cycles, indicating that the AC/PS + PD(MnO_2_/Ni) device has good electrochemical stability and that its capacitance can be well retained. The deposition mode used for the MnO_2_/Ni electrode thus plays a key role in the performance of an asymmetric supercapacitor device. The excellent cycling stability might be attributed to the favorable structure of PS + PD(MnO_2_/Ni). In PS mode, the deposition occurs continuously, producing a uniform film. In PD mode, the deposition occurs discontinuously, with a break between cycles during the deposition process, leading to more porous deposits than those obtained with PS mode. With PS deposition (+0.6 V (*vs.* SCE) for 900 s) followed by PD deposition (+0.3 and +0.6 V (*vs.* SCE)), a hierarchical porous structure is obtained (*i.e.*, PS + PD(MnO_2_/Ni)). For the AC/PS + PD(MnO_2_/Ni) device, the hierarchical porous structure and relatively high surface area provide numerous electroactive sites for the electrochemical reaction. The hierarchical pores in the AC/PS + PD(MnO_2_/Ni) device not only serve as a reservoir for the electrolyte, but also enhance the ion transportation and proton diffusion kinetics in the interior of the electrode. The porous structure can effectively mitigate the volume change during the high rate of insertion and extraction of Li^+^ ions, which can stabilize the integrity of the positive electrode, thus improving device cyclability [39,40].

Table 3 shows a comparison of the performance of the AC/PS + PD(MnO_2_/Ni) composite electrodes obtained in this study with the supercapacitors from the literature. Li *et al.* [4] prepared graphene sheet-carbon nanotube (GS-CNT) substrate and α-MnO_x_ was deposited on the substrate to be α-MnO*_x_*/GS-CNT electrodes. α-MnO*_x_*/GS-CNT exhibited very high specific energy and specific power (*ca.* 46.2 Wh·kg^−1^ and 33.2 kW·kg^−1^, respectively). Wang *et al.* [41] prepared TiO_0.54_N_0.46_ through nitriding the titanate nanotube arrays under ammonia flow at 700 °C and loaded MnO_2_ nanolayers on the TiO_0.54_N_0.46_. MnO_2_/TiO_0.54_N_0.46_ exhibited a high power density of 620 kW·kg^−1^ at an energy density of 9.8 Wh·kg^−1^. However, high cost of graphene sheet-carbon nanotube and high-temperature of nitriding process could become a major barrier for the commercialization. Lin *et al.* [20] have designed an asymmetric supercapacitor containing LiMn_2_O_4_ (cathode) and MnFe_2_O_4_ (anode). The specific energy and power density, based on the total mass of two electrodes, were 10 and 5.5 Wh·kg^−1^ at 0.3 and 1.8 kW·kg^−1^, respectively. However, LiMn_2_O_4_ and MnFe_2_O_4_ powders were mixed with a conductive agent and a polymer binder into a paste, which is then coated onto a substrate as an electrode; the overall capacity and the volumetric capacity of the electrode are significantly sacrificed due to the usage of large amounts of binder and conductive agent during electrode fabrication. Yang *et al.* [30] fabricated AC/MnO_2_ asymmetric supercapacitor, which MnO_2_ was fabricated via a CV electrodeposited route. A power density of 0.178 kW·kg^−1^ and an energy density of 37.22 Wh·kg^−1^ were reached. Unfortunately, its capacitance decreased to 80% after 1000 cycles at 2.5 mA·cm^−2^. Based on energy density and power density, the performance of AC/PS + PD(MnO_2_/Ni) device is comparable to those of MnFe_2_O_4_/LiMn_2_O_4_.

## 4. Conclusions

Nanostructured porous MnO_2_/Ni foam composite electrodes were fabricated via PD, PS, and PS + PD electrodeposition modes. SEM results indicate that the deposition mode significantly affects the morphology of MnO_2_ films. PS + PD(MnO_2_/Ni) electrodes had higher specific area, pore volume, and pore size than those of PD(MnO_2_/Ni) and PS(MnO_2_/Ni). PS + PD(MnO_2_/Ni) electrodes had a structure of interconnected macropores whose walls possess finer pores, which leads to increased mass transport through the former and high specific surface area due to the latter, giving it the highest specific capacitance among the three composite electrodes. A larger electrode/electrolyte contact area and a shorter diffusion length of Li^+^ ions can be obtained in the hierarchical porous structure of the PS + PD(MnO_2_/Ni) electrode, leading to lower inner resistance, which is beneficial for higher specific capacity. A AC/PS + PD(MnO_2_/Ni) asymmetric supercapacitor device displayed an energy density of 7.66 Wh·kg^−1^ at a power density of 600 W·kg^−1^. The capacitance retention was 98% after 10,000 cycles, indicating excellent cycling stability. The high capacitance and superior cycling stability of the AC/PS + PD(MnO_2_/Ni) asymmetric supercapacitor device can be ascribed to the synergic effect of appropriate deposition mode and Ni foam. The 3D network-like architecture of Ni foam can enlarge the electrolyte-MnO_2_ interfacial area. By using the PS + PD electrodeposition mode, a hierarchical porous structure with a high surface area of MnO_2_ grown on the backbone of Ni foam (current collector) without a polymer binder can be directly used as the electrode of a supercapacitor. Such a design can provide the 3D reticular configuration of the Ni support; moreover, the hierarchical pores not only enhance the ion transport and proton diffusion kinetics in the interior of the electrode, but also effectively mitigate volume change during repeated charge/discharge cycling.

## Figures and Tables

**Table 1:** Textural properties of PD(MnO_2_/Ni), PS(MnO_2_/Ni), and PS + PD(MnO_2_/Ni).

Sample	*S*_BET_ (m^2^·g^−1^)	*V*_pore_ (cm^3^·g^−1^)	*D*_p_ (nm)
PD(MnO_2_/Ni)	21	0.04	8
PS(MnO_2_/Ni)	86	0.30	14
PS + PD(MnO_2_/Ni)	103	0.34	13

*S*_BET_: specific surface area; *V*_pore_: pore volume; *D*_p_: pore diameter.

**Table 2:** Values of specific energy and specific power of AC/(MnO_2_/Ni) asymmetric supercapacitors.

Asymmetric Supercapacitor	Specific Energy Density (Wh·kg^−1^)	Specific Power Density (W·kg^−1^)
AC/PD(MnO_2_/Ni)	0.78	700
AC/PS(MnO_2_/Ni)	1.00	679
AC/PS + PD(MnO_2_/Ni)	9.05	708

**Table 3:** Composite metal oxides used for supercapacitors from the literature and our work.

Materials	Energy Density (Wh·kg^−1^)	Power Density (kW·kg^−1^)	Reference
α-MnO*_x_*/GS-CNT	46.2	33.2	[4]
MnO_2_/TiO_0.54_N_0.46_	9.8	620	[41]
MnFe_2_O_4_/LiMn_2_O_4_	5.5	1.8	[20]
AC/MnO_2_	37.22	0.178	[30]
AC/PS + PD(MnO_2_/Ni)	9.05	0.71	This work
